# Using simultaneous amplification and testing method for evaluating the treatment outcome of pulmonary tuberculosis

**DOI:** 10.1186/s12879-018-3424-y

**Published:** 2018-10-11

**Authors:** Liping Yan, Heping Xiao, Qing Zhang

**Affiliations:** Department of Tuberculosis, Shanghai Pulmonary Hospital, Tongji University School of Medicine, 507 Zhengmin Road, Shanghai, 200433 People’s Republic of China

**Keywords:** SAT-TB assay, Pulmonary tuberculosis, Culture test, Smear test, Tuberculosis treatment

## Abstract

**Background:**

To evaluate the utility of Simultaneous Amplification and Testing (SAT-TB) Method for monitoring anti-TB treatment response.

**Methods:**

Serial morning sputum specimens were obtained from 377 active pulmonary tuberculosis (PTB) cases at baseline, weeks 2, months 2, 5 and 6 (newly diagnosed patients) or 8 (previously treated patients) for AmpSure assay, smear fluorescence microscopy (FM) and BACTEC MGIT 960 culture assay.

**Results:**

After treatment of 2 weeks, sputum culture was positive in 280 patients (74.27%). Among whom, 219 patients tested positive for SAT-TB assay and 143 patients smear FM positive. The detection rate of SAT-TB (78.21%) was significantly higher than sputum FM (51.07%, χ2 = 45.128, *P* < 0.001). At the end of the second month of treatment, 157 patients (41.64%) were still culture-positive, 115 patients of them SAT-TB positive and 79 smear FM positive. The difference of detection rate between SAT-TB (73.25%) and sputum FM (50.32%) was significant (χ2 = 17.480, *P* < 0.001). When patients underwent five months of treatment, 65 patients (17.24%) with sputum culture positive was defined as treatment failure. Among whom, 60 patients (92.31%) were SAT-TB positive and 38 patients (58.46%) were smear FM positive. The detection rate of SAT-TB assay was significantly higher than sputum FM (χ2 = 17.333, *P* < 0.001).

**Conclusion:**

Results of AmpSure assays for monitoring treatment responses can be obtained without waiting for the results of BACTEC MGIT 960 assays and most patients with treatment failures could be detected after 5 months.

## Background

Tuberculosis (TB) is the leading cause of death from a single infectious disease [1]. Drug resistance further aggravates the problem. Effective drug treatment of active pulmonary tuberculosis (PTB) patients is critical for TB control. New TB patients are treated with 2HRZE/4HR regimen according to recommendation of World Health Organization (WHO) and 2HRZES/6HRE regimen are used in previously treated patients with drug-susceptible pulmonary TB [2]. All patients in the treatment of TB should be monitored to evaluate their response to treatment by sputum examination. Monitor the patient by chest radiography is not reliable and wasteful of resources. Sputum culture conversion (SCC) is commonly used as a microbiological endpoint in the treatment of tuberculosis [3]. 2-month, 5-month and 6-month or 8-month SCC status is proxy marker for treatment outcome. However, Sputum culture is not convenient in routine clinical practice [4]. It will take weeks to obtain the results. Smear microscopy is widely used in most undeveloped countries. Sputum smear is performed at the end of the 2nd month (the intensive phase of treatment), the 5th month and treatment completion [5]. If smear is positive at month 2, sputum smear is repeated at month 3. If smear is positive at month 2 or at month 5, sputum culture and drug susceptibility testing (DST) should be obtained [5]. Smear positive at the end of the 2nd month indicates the patient may have drug-resistant M. tuberculosis. Smear-positivity at the end of the fifth month is defined as treatment failure and it is necessary to change the present treatment regimen. However, its sensitivity being unacceptably flawed [6] and non-viable bacteria remain visible by microscopy. In addition, microscopy could not distinguish tuberculos mycobacteria (NTM) from Mycobacterium tuberculosis complex (MTBC) [5].

An increasing number of molecular methods, that is more rapid and sensitive than existing conventional tests, have become available [7–9]. Simultaneous amplification and testing methods for detection of MTBC (SAT-TB assay), that is based on nucleic acid isolation, real-time fluorescence simultaneous isothermal RNA amplification and fluorescence-labeled hybridization probes testing has the advantage of rapid, simple and good reproducibility. The results could be obtained within 120 min.

Moreover, the SAT-TB assay is intended for point-of-care testing for TB [10]. Recent researches showed that sensitivity of the SAT-TB assay for the diagnosis of PTB with sputum samples was from 39.2 to 93% [11, 12]. In patients with sputum-scarce, the sensitivity of the SAT-TB test using bronchial alveolar lavage fluid (BALF) specimens was 50.75% [13]. However, studies using the SAT-TB assay to evaluate anti-TB treatment outcome have not been reported.

For the above reasons, we designed a prospective study to investigate the efficacy of SAT-TB assays for monitoring response to anti-TB therapy with sputum specimens from new PTB patients and previously treated patients in China.

## Methods

### Patients

This prospective study was approved by The Ethics Committee of the Shanghai Pulmonary Hospital. Each participant gave written informed consent before enrollment.

We prospectively screened all confirmed active PTB patients based on culture positive in Shanghai Pulmonary Hospital from January 2016 to January 2017. Informations about sex; age; TB contacts; symptoms of TB; history of anti-TB treatment; comorbidities and concurrent therapies were routinely collected from each patient by attending physicians using a questionnaire. The standard 2HRZE/4HR regimen are used in newly PTB patients according to recommendation of WHO and 2HRZES/6HRE regimen in previously treated patients under direct observed treatment (DOT) strategy [2]. The exclusion criteria included: (1) HIV test positive; (2) aged more than 17 years; (3) inability to provide sputum for examinations; (4) with concomitant extra-pulmonary TB; (5) resistant to any drug of the two regimens; (6) participate in other clinical researches at the same time; (7) had a history of poor adherence in other clinical trials before.

### Examinations

Morning sputum specimens were obtained from enrolled patients at baseline (at the time of diagnosis before initiation of the treatment) (T0) and at the end of 2-week (T1), 2-month (T2), 5-month (T3) and 6-month (initially diagnosed patients) or 8-month (previously treated patients) (T4) after treatment initiation. M. tuberculosis detection was performed using smear fluorescence microscopy (FM), bacteriological analysis and the AmpSure assay. Smear positive grade 1 was the detection threshold of a positive sputum smear. Bacteriological analysis was done using the BACTEC MGIT 960 System (Becton Dickinson Diagnostic Systems, Sparks, MD, USA), according to WHO guidelines [14]. The SAT-TB assay was performed by means of the AmpSure assay (Shanghai Rendu Biotechnology, Shanghai China) according to the protocol of manufacturer, which was described previously [15]. Initial MTB isolates obtained from patients at the time of 2-month were subjected to DST for ofloxacin (Ofx),

Isoniazid (H), rifampicin (Rfp), ethambutol (E), streptomycin (Sm), amikacin (Am), and capreomycin (Cm) using BACTEC MGIT 960 System according to WHO guidlines [14]. All of the above tests were performed in a tuberculosis reference laboratory of Shanghai Pulmonary Hospital. Quality control was performed routinely following the Manual of procedures of the National Tuberculosis Control program. Sputum specimens were given identification laboratory numbers, and technicians who carried out bacteriological investigations were blinded to the patients.

Computed tomography (CT) scans was taken on all study participants at baseline, months 2 and the end of the course or anytime if necessary. Two radiologists and a physician independently evaluated all images. A standard format was followed to make sure the radiological readings were optimized for interpretation.

### Definitions

End-of-treatment outcomes were assigned according to WHO definitions [3], namely, cure: a negative sputum smear or culture result in the last month of the therapy and on previous occasion at least once; treatment completed: completed the therapy but without a sputum smear- or culture- result in the last month of the therapy and on previous occasion at least once; failure: have a positive sputum smear or culture result at the 5th month or later or a multidrug-resistant (MDR) strain was found at anytime during the treatment; died: died due to any cause during the treatment; default: treatment was suspended for ≥2 consecutive months; transfer out: transferred to other registration department. Cure or treatment completion was defined as treatment success [3].

MDR is defined as resistance to at least isoniazid and rifampin. Extensively drug-resistant tuberculosis (XDR-TB), by definition, is a form of MDR-TB plus resistance to at least one drug in both of the two most significant groups of drugs in an MDR-TB schedule: fluoroquinolones (FQs) and second-line injectable agents (amikacin, capreomycin or kanamycin). Pre-XDR-TB is defined as MDR-TB plus resistance to either a FQs or a second-line injectable agent.

### Statistical analyses

SPSS 18.0 software for Windows (Version 18.0, SPSS Inc., Chicago) was used to analyze the data. Numerical variables were shown as the mean ± standard deviation. Fisher exact test or Pearson chi-squared analysis was used to analyze categorical variables. *P* ≤ 0.05 was set as the level of statistical significance.

## Results

**Fig. 1:**
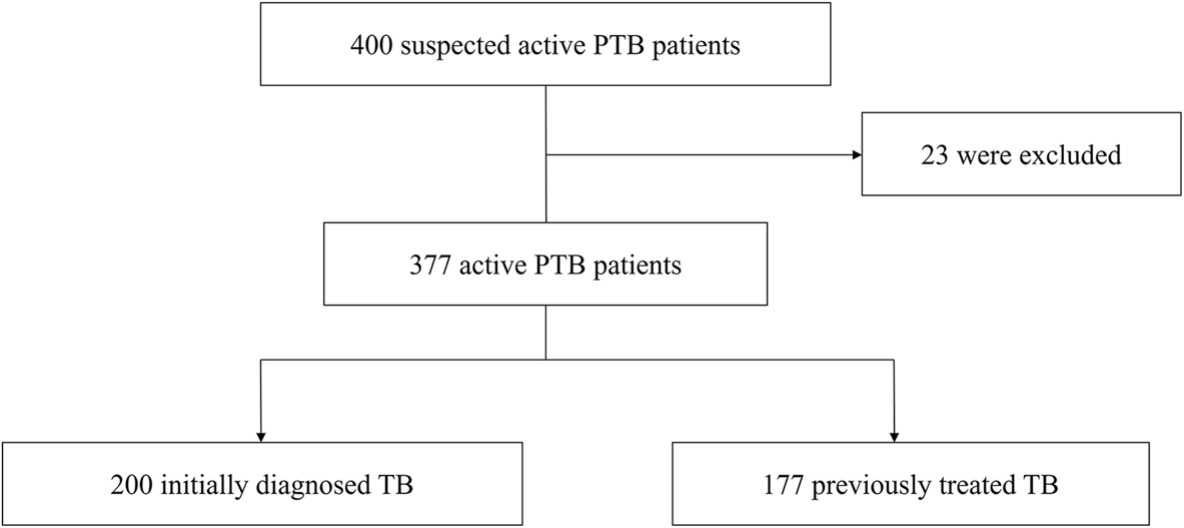
Flow chart of the study

We enrolled 400 suspected active PTB patients, prospectively. Twenty-three of them were excluded from our analysis because of NTM. The remaining 377 confirmed patients including 200 initially diagnosed TB cases and 177 previously treated cases with a mean age of 46.4 ± 17.5 years were eligible for this analysis and were followed during the standard anti-TB treatment (Fig. 1). These patients were predominantly males (69.5%) with a mean body mass index (BMI) of 19.0 kg/m^2^. The participants had typical CT findings that suggested active TB, including cavitary lesions, tree-in-bud appearance and lung nodules. There are 1820 sputum specimens collected for examinations. The demographic and clinical characteristics of 377 patients were summarized in Table 1.

**Table 1:** Patient characteristics

Variable	New PTB (*n* = 200)	Previously treated (*n* = 177)	Total (*n* = 377)
Men (n [%])	135 (67.5)	127 (71.75)	262
Women (n [%])	65 (32.5)	50 (28.25)	115
Age (y) (median) (range)	44.6 ± 17.7	47.4 ± 19.7	46.4 ± 17.5
Body mass index (median) (range)	19.1 (13–27)	18.9 (13–27)	
Cured cases	173	139	312
Failed cases	27	38	65

After treatment of 2 weeks, sputum culture was positive in 280 patients (74.27%). Among whom, 219 patients tested positive for SAT-TB assay and 143 patients smear FM positive. The detection rate of SAT-TB (78.21%) was significantly higher than sputum FM (51.07%, X^2^ = 45.128, *P* = 0.000). At the end of the second month of treatment, 157 patients (41.64%) were still culture-positive, 115 patients of them SAT-TB positive and 79 smear FM positive. The difference of detection rate between SAT-TB (73.25%) and sputum FM (50.32%) was significant (X2 = 17.480, *P* = 0.000). When patients underwent five months of treatment, 65 patients (17.24%) with sputum culture positive was defined as treatment failure. Among whom, 60 patients (92.31%) were SAT-TB positive and 40 patients (61.54%) were smear FM positive. The detection rate of SAT-TB assay was significantly higher than sputum FM (X2 = 17.333, *P* < 0 .001). Ultimately, 312 patients (82.76%) cured with culture clearing and imaging improvement. The results of SAT-TB assay, smear FM, and BACTEC MGIT 960 culture at different time points were summarized in Table 2.

**Table 2:** Summary of SAT-TB, smear FM, and culture results

		SAT-TB (+)		SAT-TB (−)	
		Smear (+)	Smear (−)	Smear (+)	Smear (−)
before treatment	Mt-culture (+)	236 (62.6%)	134 (35.55%)	0	7 (1.86%)
	Mt-culture (−)	0	0	0	0
2nd week	Mt-culture (+)	143 (51.07%)	76 (27.15%)	0	61 (21.78%)
	Mt-culture (−)	0	0	0	97 (25.73%)
2nd month	Mt-culture (+)	79 (50.32%)	36 (22.93%)	0	42 (26.75%)
	Mt-culture (−)	0	0	0	220 (59.46%)
5th month	Mt-culture (+)	38 (58.46%)	22 (33.84%)	0	5 (7.7%)
	Mt-culture (−)	0	0	2 (0.65%)	310 (99.35%)
6th/8th month	Mt-culture (+)	0	0	0	0
	Mt-culture (−)	0	0	0	312 (82.76%)

**Fig. 2:**
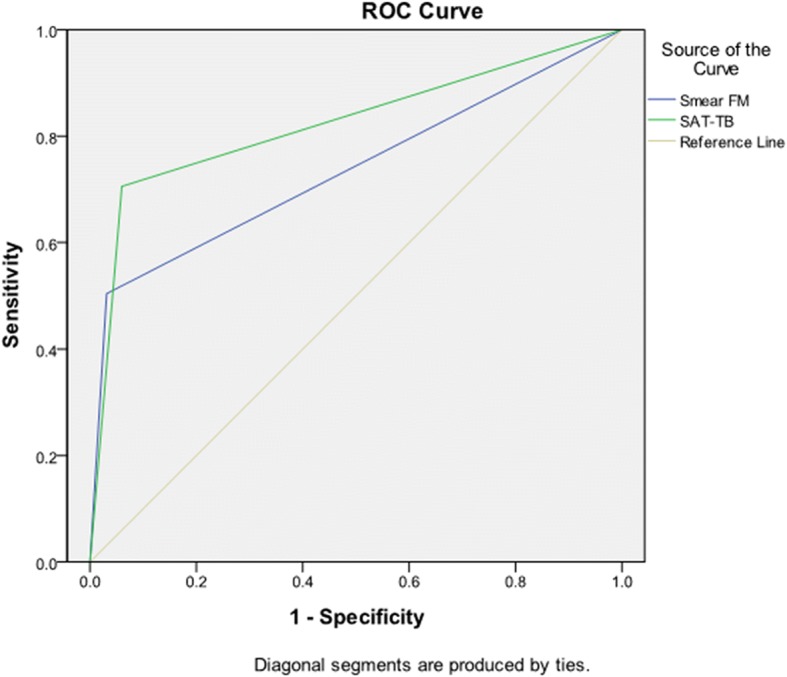
ROC curve for SAT-TB and smear FM

The overall sensitivity of SAT-TB assay was 86.92%, which was significantly higher than that of smear FM (56.43%) (*P* < 0.001). The overall specificity of SAT-TB assay was 100%, which was also higher than that of smear FM (99.69%) (*P* = 0.156). The PPV and NPV of SAT-TB assay was 100% and 84.55%, respectively. The PPV and NPV of smear FM was 99.6% and 62.28%, respectively. The difference between PPV had no statistical significance (*P* = 0.080), but the difference between NPV had statistical significance (*P* < 0.001) (Fig. 2).

Initial MTB isolates obtained from positive culture of 157 patients at the end of the second month of treatment were subjected to DST. The DST results were summarized in Table 3. There were 6 XDR-TB, 9 pre-XDR-TB, 32 MDR-TB and 6 mono-drug resistant (resistant to streptomycin) TB patients. All of the 47 MDR/XDR-TB and 3 mono-drug resistant as well as 15 susceptible TB patients failed.

**Table 3:** Summary of drug susceptibility testing results of 157 patients

		XDR	Pre-XDR	MDR	Sm-resistant	Susceptible	Total
New PTB	cure	0	0	0	1	34	35
	failure	0	2	9	1	15	27
Previously treated	cure	0	0	0	2	55	57
	failure	6	7	23	2	0	38
Total		6	9	32	6	104	157

## Discussion

In the current study, we used SAT-TB assay to evaluate treatment outcome in Chinese PTB patients compared with smear FM and BACTEC MGIT 960 at different time points, and observed that the SAT-TB assay is preferred. All the patients that was in the treatment of TB should be monitored the response to treatment. If sputum smear is positive at month 2, sputum smear is repeated at month 3. If smear is positive at month 3, sputum culture and DST should be obtained. Smear- positivity or culture-positivity at the end of the fifth month is defined as treatment failure [3]. Unfortunately, sputum smear and culture have significant limitations. The technological development of nucleic acid amplification has led to advances in the early detection of tubercle bacilli. The GeneXpert assay (Cepheid, Sunnyvale, CA) was introduced currently in China. The high price of the GeneXpert assay and the requirement of costly especial instrument for the MTD test (Gen-Probe; San Diego, CA, USA) prevent these assays from being a routine test in resource-limited countries [16, 17]. The SAT-TB assay has another advantage over PCR, as the detection objective is rRNA. RNA is much more unstable than DNA, so a positive result indicates the presence of viable MTBC. Therefore the false positive rate decreased. In addition, the SAT-TB assay can be performed on real-time PCR instruments, which can be found in most clinical laboratories.

The SAT-TB assay was demonstrated to have better sensitivity and high specificity for the detection of tubercle bacilli in our study. Over the intensive phase of therapy, 73.25% of culture-positive patients could be detected with the SAT-TB assay within 2 h. Month-2 culture conversion is generally used as substitute index of anti-TB treatment efficiency [18]. Detection of tubercle bacilli at the end of the intensive phase remains important as it may indicate that the patient may have multidrug-resistant M. tuberculosis and DST should be performed. If we use sputum smear for monitoring the treatment response, only about half of culture-positive patients (50.32%) could be detected. If we use the SAT-TB assay, DST could be performed in three-quarters of these patients. The number of patients in need of DST was increased by about a quarter. Drug-resistant tuberculosis (DR-TB) should be identified as early as possible not only for achieving the best treatment outcome but also for controlling the spread of DR-TB. Smear-positivity at the end of the fifth month is defined as treatment failure and it is necessary to change the present treatment regimen. In this study, 65 patients with sputum culture positive were defined as treatment failure. Among whom, 60 patients were SAT-TB assay positive. This means that if we use the SAT-TB assay for monitoring the treatment response, 92.31% of patients with treatment failure can be found timely and changed to appropriate therapy. However, only 58.46% of these culture-positive patients were sputum smear positive. After a month or three, physicians may mistakenly declare the treatment successful for 41.54% of these failure patients. It’s worth mentioning that two sputum smear positive were identified as false-positives or laboratory contamination according to culture and SAT-TB assay negative and imaging improvement at the end of the fifth month. Ultimately, the treatment success rate in this study was 82.76%, which was comparable to the average success rate in the world. Globally, the treatment success rate for the 5.9 million new and relapse cases was 83% in 2015 [1]. False-negative findings, however, are inevitable with the current technology [19–21]. In addition, the major barrier in applying molecular detection method in evaluating treatment response is the problem of false positive rather than false negative. In our research, there was no false positive (sputum culture-negative but SAT-TB assay positive) for SAT-TB assay.

Treatment outcome of anti-TB treatment in bacteriologically confirmed PTB patients is evaluated principally by serial bacteriologic examinations [2], whereas Treatment outcome in clinically diagnosed PTB patients are usually monitored clinically or radiographically. To overcome these shortcomings, numerous biomarkers have been evaluated [4, 22], although most of the markers do not have high levels of validity. More and more studies confirmed that the possible function of plasma-based IFN-γ-release assays (IGRAs) for evaluation of reaction to anti-TB therapy is objectionable [23]. The IGRA is less useful in terms of treatment monitoring, given that the IGRA is based on the TB antigen-stimulated immunologic reaction, which tends to persist and shows blunted fluctuations during the longitudinal monitoring of TB [24]. Lago et al. observed a consistent decrease in IL-10 levels in active PTB patients at all times of therapy, suggesting that this cytokine could be used as a helpful biomarker for evaluating disease progression [25]. Therefore, novel accessing means of anti-TB therapy outcome are required to make anti-TB therapy better in some clinical circumstances.

In this study, 65% of patients were male. Men seem to be more affected with TB than women with a male/female ratio of 1.86 for the worldwide case notification rate in 2017 [26]. This excess of male pulmonary TB cases is seen in almost all countries of the world, and this gender effect is thought to be related to many factors, such as access to healthcare, nutritional status, socioeconomic and cultural factors. TB disease is not gender specific. Male and female are equally susceptible.

There are several limitations in this study. First, the number of patients with microbiological diagnosed active TB is relatively small. Second, this study was limited to only HIV-negative PTB patient group, which limits its generalization to the HIV-infected group. HIV-positive cases, a clinically significant population, were removed from this study as they must be shifted to special hospitals in China. Third, a few variables such as chest imaging manifestations were not explored in this analysis. Finally, culture after treatment was not followed up in this study. Despite these shortcomings, as far as we know, this is likely to be the first prospective study to evaluate the utility of SAT-TB assays to access TB therapy outcome.

## Conclusions

In summary, our research suggests that the SAT-TB assay is preferred to evaluate treatment outcome in Chinese PTB patients compared with smear FM and BACTEC MGIT 960 at different time points. Results of AmpSure assays for monitoring treatment responses can be obtained without waiting for the results of BACTEC MGIT 960 assays and most patients with treatment failures could be detected after 5 months. Whether SAT-TB assays can be used to access TB therapy outcome needs to be explored further.

## Abbreviations

Am: Amikacin
BALF: Bronchial alveolar lavage fluid
Cm: Capreomycin
CT: Computed tomography
DOT: Direct observed treatment
DR-TB: Drug-resistant tuberculosis
DST: Drug susceptibility testing
E: Ethambutol
FM: Fluorescence microscopy
FQs: Fluoroquinolones
H: Isoniazid
IGRAs: IFN-γ-release assays
MDR: Multidrug-resistant
MTBC: Mycobacterium tuberculosis complex
NTM: Non- tuberculos mycobacteria
Ofx: Ofloxacin
PTB: Pulmonary tuberculosis
Rfp: Rifampicin
SAT-TB assay: Simultaneous amplification and testing methods for detection of MTBC
SCC: Sputum culture conversion
Sm: Streptomycin
TB: Tuberculosis
WHO: World Health Organization
XDR-TB: Extensively drug-resistant tuberculosis
